# Neural reflex regulation of systemic inflammation: potential new targets for sepsis therapy

**DOI:** 10.3389/fphys.2014.00489

**Published:** 2014-12-15

**Authors:** Ricardo Fernandez, Gino Nardocci, Cristina Navarro, Edison P. Reyes, Claudio Acuña-Castillo, Paula P. Cortes

**Affiliations:** ^1^Laboratorio de Fisiología, Departamento de Ciencias Biológicas, Facultad de Ciencias Biológicas y Facultad de Medicina, Universidad Andrés BelloSantiago, Chile; ^2^Centro de Fisiología Celular e Integrativa, Facultad de Medicina, Clínica Alemana – Universidad del DesarrolloSantiago, Chile; ^3^Dirección de Investigación, Universidad Autónoma de ChileSantiago, Chile; ^4^Departamento de Biología, Facultad de Química y Biología, Universidad de Santiago de ChileSantiago, Chile; ^5^BioAdvisingSantiago, Chile

**Keywords:** systemic inflammation, sepsis, reflex control of inflammation, carotid body, vagus nerve

## Abstract

Sepsis progresses to multiple organ dysfunction due to the uncontrolled release of inflammatory mediators, and a growing body of evidence shows that neural signals play a significant role in modulating the immune response. Thus, similar toall other physiological systems, the immune system is both connected to and regulated by the central nervous system. The efferent arc consists of the activation of the hypothalamic–pituitary–adrenal axis, sympathetic activation, the cholinergic anti-inflammatory reflex, and the local release of physiological neuromodulators. Immunosensory activity is centered on the production of pro-inflammatory cytokines, signals that are conveyed to the brain through different pathways. The activation of peripheral sensory nerves, i.e., vagal paraganglia by the vagus nerve, and carotid body (CB) chemoreceptors by the carotid/sinus nerve are broadly discussed here. Despite cytokine receptor expression in vagal afferent fibers, pro-inflammatory cytokines have no significant effect on vagus nerve activity. Thus, the CB may be the source of immunosensory inputs and incoming neural signals and, in fact, sense inflammatory mediators, playing a protective role during sepsis. Considering that CB stimulation increases sympathetic activity and adrenal glucocorticoids release, the electrical stimulation of arterial chemoreceptors may be suitable therapeutic approach for regulating systemic inflammation.

## Inflammatory response and its regulation

Inflammation can be defined as a “host defense in response to injury of vascularized tissues” (Majno and Joris, 1996). The inflammatory response –the first alert to the signals that address perturbation– involves an innate system of cellular and humoral responses that, following injury, will support the organism in attempts to restore tissue homeostasis (Chaplin, 2010). The inflammatory response involves a complex network of events involving several organizational levels: at the systemic level, the leakage of substances from the vascular compartment (e.g., water, salt, and proteins); at the cellular level, the activation of endothelial cells and macrophages, leukocyte-endothelium adhesive interactions, and the recruitment of leukocytes; at the subcellular level, the activation and aggregation of platelets, the release of proteases and oxidants from phagocytic cells, and the activation of the complement, clotting and fibrinolytic systems. All the above-mentioned events may assist in addressing a state of injury (Alvarez Perez Gil et al., 2012). During the inflammatory response, the number of inflammatory mediators found in the plasma and secreted by cells is broad and includes extracellular Danger-Associated Molecule Patterns (DAMPs), such as cellular debris, potassium, DNA, cytokines, histones and high-mobility group protein B1 (HMGB1), and Pathogen-Associated Molecular Patterns (PAMPs), such as bacterial lipopolysaccharide (LPS, endotoxin) or peptidoglycan, fungal zymosan or viral single stranded RNA (Deutschman and Tracey, 2014). DAMPs are sensed, leading to the assembly of the inflammasome, and DAMPs and PAMPs catalyze the formation of cell surface signalosomes. Both signalosomes and inflammasomes induce apoptosis, intracellular stress and other metabolic responses, such as the expression of pro-inflammatory cytokines (e.g., interleukin (IL)-1β, IL-6, tumor necrosis factor (TNF)-α, and HMGB1), cell proliferation and the amplification of the innate immune response (Lamkanfi et al., 2010).

Reversal or resolution of the inflammatory response implies that leukocytes are removed, either via lymphatics or apoptosis, and that the ongoing acute inflammatory response is terminated. As a consequence, during resolution, increased vascular permeability is reversed, and immune cell emigration from the blood compartment ceases (Rock et al., 2010).

Therefore, to maintain immunological homeostasis, inflammation resolution is essential for inhibiting the release and toxicity of potentially damaging inflammatory mediators (Nathan and Ding, 2010). Moreover, regulatory T cells, alternatively activated macrophages and other cellular mechanisms also suppress excessive immune responses to prevent tissue damage. Numerous anti-inflammatory factors, such as cytokines (e.g., IL-4, IL-10, and transforming growth factor (TGF)-β), protease inhibitors, and antioxidant enzymes, which are present in plasma at very low concentrations, are induced by the immune challenge and are powerful anti-inflammatory factors comprising contain the acute inflammatory response. As a result, these regulatory factors decreased the production of pro-inflammatory mediators and reduce the number of immune cells accumulating in tissues (Buras et al., 2005). Nevertheless, there are several limitations of these humoral and cellular mechanisms: (i) they are slow compared to environmental changes; (ii) they are unable to integrate biological responses to numerous stimulating inputs across a network of immune tissues; and (iii) these mechanisms rely on the circulatory system and thus are anable to respond efficiently in a specific tissue or confined region (Andersson and Tracey, 2012).

**Figure 1:**
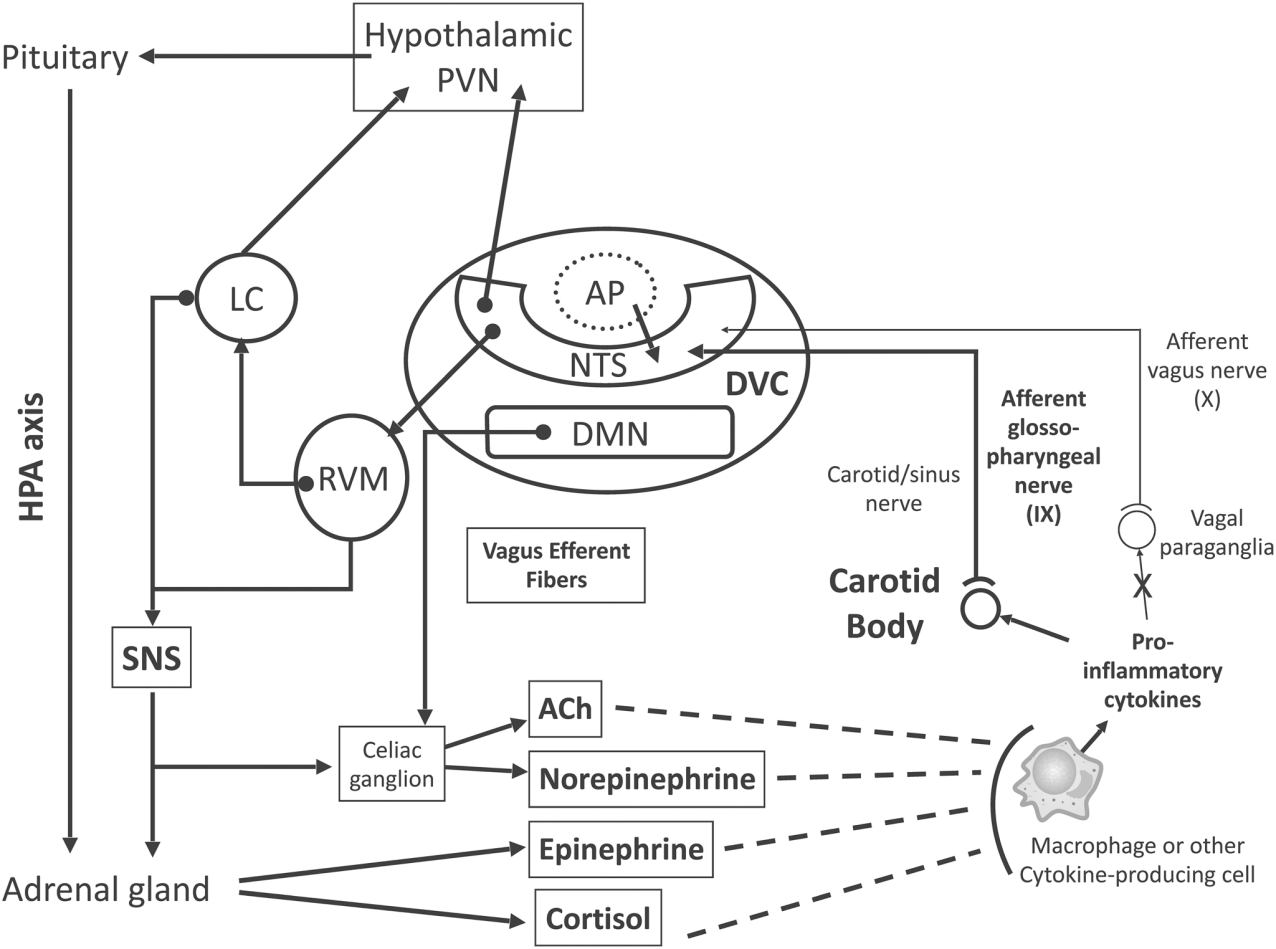
**Proposed model for inflammation-induced central nervous system (CNS) activation through carotid body (CB) chemoreceptors and subsequent efferent neuroimmunomodulator activation**. Briefly, chemosensory transduction begins in immune cells, which release inflammatory mediators (i.e., TNF-α, IL-1β, and IL-6) to activate the CB. The CB is innervated by glossopharyngeal (cranial nerve IX) afferent neurons, the cell bodies of which are located in the petrosal ganglion, and their central projections end primarily within the dorsal vagal complex (DVC) of the *medulla* oblongata. The DVC consists of the *nucleus tractus solitarii* (NTS), the dorsal motor nucleus of vagus nerve (DMN), and the *area postrema* (AP). The DMN is the main site of origin of preganglionic vagus efferent fibers, which evoke acetylcholine (ACh) release from celiac ganglion neurons, inhibiting the pro-inflammatory response. The AP lacks a brain-blood barrier (dotted line, AP), projects into the NTS and is an important site for humoral immune-to-brain communication. The main portion of the CB chemosensory inputs is received by neurons in the NTS, which coordinate autonomic function and interaction with the endocrine system. Ascending projections from the NTS reach the hypothalamic paraventricular nucleus (PVN), an important structure involved in hypothalamus-pituitary-adrenal (HPA) axis activation, releasing cortisol, which, in fact, inhibits the pro-inflammatory response. Synaptic contacts also exist between neurons in the NTS and rostral ventrolateral *medulla* (RVM), which plays an important role in the control of cardiovascular and respiratory homeostasis. Neurons from the RVM project to the *locus coeruleus* (LC), which innervates higher brain sites, such as the PVN. Neuronal projections emanate from the RVM and LC to sympathetic preganglionic neurons (SNS) in the spinal cord, which innervates both the adrenal *medulla* and celiac ganglion for epinephrine and norepinephrine release, respectively, inhibiting the pro-inflammatory response. These ascending and descending connections provide a neuronal substrate for neuroimmunomodulatory mechanisms. Excitatory pathways, continuous lines; inhibitory pathways, dashed lines.

An increasing amount of research shows that neural signals play a significant role in modulating the immune response (Glaser and Kiecolt-Glaser, 2005). In fact, both immune-suppression and immune-enhancement can be behaviorally modified in experimental animals (Cohen et al., 1994). Therefore, the central nervous system (CNS) communicates with the immune system and regulates it. Several pathways allow the CNS to regulate the transcription of immune response genes in peripheral tissues (Figure 1): (i) the hypothalamic–pituitary–adrenal (HPA) axis and the production of glucocorticoids, which suppress pro-inflammatory immune response genes (Herman et al., 2012); (ii) the sympathetic nervous system (SNS), which innervates primary and secondary lymphoid organs, the vasculature and peripheral organs and tissues, acting through norepinephrine to modify hematopoiesis and the interactions between antigen-presenting cells and lymphocytes (Nance and Sanders, 2007); (iii) SNS-induced epinephrine release by the adrenal gland, which upregulates the transcription of pro-inflammatory cytokines (Cole et al., 2007); (iv) the activation of the cholinergic anti-inflammatory system, whereby parasympathetic (vagal) outflow arrives at the celiac ganglion, and then releases acetylcholine (ACh) through cholinergic fibers from the splenic nerve, attenuating spleen cytokine production (Borovikova et al., 2000; Tracey, 2002; Rosas-Ballina and Tracey, 2009); (v) the local (peripheral tissues) release of physiological neuromodulators such as pain-related neuropeptides and enteric system-regulating neuropeptides, and circulating mediators, such as endogenous opioids, insulin-like growth factor, growth hormone, and other hormones (e.g., prolactin, Freeman et al., 2000) that can affect the innate and adaptive immune system (Irwin and Cole, 2011).

It is clear that in additionto neural reflexes maintaining homeostasis in other body systems, neural circuits also regulate immunity. Therefore, what is(are) the afferent pathway(s) by which the immune system relay signals to the central nervous system?

## Systemic inflammation and sepsis

Although inflammation is mostly a beneficial host response to foreign challenge or tissue injury, leading to the restoration of tissue structure and function, it can contribute to the pathogenesis of many diseases. Systemic inflammation results from the dysregulation of local inflammation when the host is unable to contain an insult, regardless of whether it is caused by bacteria, trauma, burn, or drug overdose. Sepsis is defined as “the systemic inflammatory response syndrome that occurs during infection” (Bone et al., 1992) and is mainly due to host cell stimulation (monocytes/macrophages, endothelial, and polymorphonuclear cells) to produce and release pro-inflammatory cytokines (Schletter et al., 1995). Sepsis involves the evidence of infection and may be associated with fever, tachycardia, tachypnea, altered white blood cell count, and decreased arterial oxygen partial pressure. Sepsis progression to severe sepsis and septic shock involves many pathological processes including hemodynamic abnormalities (e.g., hypoperfusion or hypotension) and also involves oliguria, lactic acidosis, acute alteration in mental state and multiple organ dysfunction (MOD) syndrome (Riedemann et al., 2003).

However, immunological mechanisms do not completely explain the basis of cellular dysfunction and MOD. Indeed, systemic inflammation affects several systems within the body, including metabolic, hormonal, and neural pathways (Singer et al., 2004; Carre and Singer, 2008). Thus, systemic inflammation initiates the disruption of communication between different organ systems, and subsequently, MOD reflects a progressive uncoupling that may become permanent. With an increasing projected incidence of 1.5% *per annum* in the United States, and an average cost per case of US$22,100 (Angus et al., 2001), sepsis syndromes and MOD are the main cause of death of critical care patients because despite many efforts and significant advances in maintaining therapies (Martin et al., 2003), there is no particularly effective therapy for these conditions (Riedemann et al., 2003). Thus, the knowledge of immunometabolic and neurophysiological mechanisms and the pathophysiology underlying sepsis progression to MOD and death could help to improve current therapies and identify new pharmacological therapeutic targets.

The pro-inflammatory cytokine TNF-α is an important mediator of the lethal effect of endotoxin (Tracey et al., 1986). In fact, reducing the activity or the expression of TNF-α significantly diminishes endotoxin-induced damage, and the degree of tissue damage can be correlated to the amount of TNF-α in serum (Yang et al., 2007). Damage may result in microvascular dysregulation and/or mitochondrial dysfunction (Crouser, 2004), which results in MOD and death. TNF-α is released during the first 30–90 min after exposure to LPS, triggering a second level of inflammatory cascades that involve other cytokines, reactive oxygen species, lipid mediators, and the up-regulation of cell adhesion molecules. Normally, the pro-inflammatory response is counter-balanced by a group of regulatory molecules, such as IL-10 (an anti-inflammatory cytokine), which attempt to restore immunological equilibrium (Scumpia and Moldawer, 2005). In fact, the main stimulus for IL-10 production is inflammation itself. Both TNF-α and IL-1β directly stimulate IL-10 production, suggesting the existence of a negative feedback loop, whereby the production of IL-10 is limited to the inflammatory process (Van Der Poll et al., 1994). Therefore, host damage can result directly by excessive inflammation, or indirectly through immune dysfunction, and host survival depends on the intensity of and the correct balance between pro- and anti-inflammatory responses.

## Reflex regulation of systemic inflammation: immune-to-brain communication

Research into immunosensory activity has been focused on the origin of signaling, i.e., plasma pro-inflammatory cytokines such as TNF-α, IL-1β, and IL-6. In fact, direct injection of these cytokines into the brain causes fever, activation of the HPA axis and sickness-like symptoms, mimicking a real immune challenge (Quan, 2014). Immune system-derived signals are conveyed to the CNS through four different pathways. The circumventricular organs (CVOs) were among the first immune-to-brain pathways proposed (Blatteis et al., 1987; Stitt, 1990). These regions have a leaky brain-blood barrier (BBB), and several are situated near the CNS areas that are known to react against peripheral immune challenges, such as the *area postrema* (AP) and the *organum vasculosum*. The former contains neurons that project into the *nucleus tractus solitarii* (NTS) (Cai et al., 1996), a well-known target of neuroimmune activation, and the latter is involved in febrile responses. Another (second) afferent pathway occurs via the saturable transport of cytokines across the BBB (Banks and Erickson, 2010), contributing to an increase in neuroinflammation. A less direct pathway (third) is the binding of cytokines to brain endothelial cells, which evokes the release of paracrine mediators such as IL-1, IL6, and prostaglandins (Fabry et al., 1993; Cao et al., 1998; Quan, 2014). Finally, the fourth pathway occurs through the activation of peripheral sensory nerves, i.e., the vagus nerve (Goehler et al., 1997).

Wan et al. showed that subdiaphragmatic vagotomy blocks brain c-fos induction after the intraperitoneal (IP) administration of LPS (Wan et al., 1994), suggesting that neural, rather than humoral, pathways are capable of transmitting inflammatory signals to the brain. However, the involvement of peripheral sensory nerves in immunomodulation is controversial. Inflammatory mediators released by immune cells are able to activate both vagal paraganglia (Goehler et al., 1997, 1999) and primary afferent neurons located in sensory ganglia, which, in turn, evokes host defense reflexes. Vagal paraganglia consist of two cell types: type I (glomus) cells and type II (sustentacular) cells (Berthoud et al., 1995). The cell bodies of vagal afferent neurons innervating vagal glomus cells (GC) are located in the nodose ganglion, and the central projections of these neurons arrive primarily at the dorsal vagal complex (DVC) of the *medulla oblongata*.

The DVC, which involves the AP, the dorsal motor nucleus of the vagus (DMN) and the NTS (Berthoud and Neuhuber, 2000), is the main site of origin of preganglionic vagus efferent fibers, whereas cardiovascular vagal efferents originate within the medullar *nucleus ambiguus* (NA). As previously mentioned, the AP is an important CVO and a putative site for humoral immune-to-brain communication. Neurons in the NTS receive the main portion of vagal sensory inputs. In turn, the NTS coordinates autonomic function and interaction with the endocrine system, and its ascending projections reach the hypothalamic paraventricular nucleus (PVN), an important structure involved in the HPA axis activation. Similarly, the NTS projects to the rostral ventrolateral medulla (RVM), which has an important function in the control of cardiovascular and respiratory homeostasis. Neurons from the RVM project to the *locus coeruleus* (LC), which innervates higher brain sites, such as the hypothalamus and PVN. Neuronal projections from the RVM and LC reach sympathetic preganglionic neurons in the spinal cord (Fernandez and Acuna-Castillo, 2012). Conversely, the PVN projects back to the RVM and NTS (Pavlov et al., 2003), completing the necessary connections for interaction between the HPA axis and autonomic (sympathetic and parasympathetic) cardiorespiratory reflex activation. Furthermore, these interactions provide a neuronal substrate for an immunomodulatory mechanism (Figure 1).

## Immunosensory signaling through the vagus nerve

As previously mentioned, subdiaphragmatic vagotomy blocks brain c-fos induction after IP administration of LPS (Wan et al., 1994). Moreover, if the procedure is performed prior to the IP injection of IL-1β, the cytokine fails to evoke a hyperthermic response (Watkins et al., 1995). Thus, abdominal LPS or IL-1β activates sensory neurons in the vagus nerve, which, in turn, activates hyperthermia-related brainstem nuclei (Quan, 2014). In contrast, food-motivated behavior is suppressed by peripheral LPS or IL-1β; this depression is also blocked by vagotomy (Bret-Dibat et al., 1995).

Vagal primary afferent fibers are stimulated by IP administration of LPS (Goehler et al., 1999, 2000), and these fibers activate the CNS neurons responsible for the manifestations of systemic inflammatory response syndrome (Mascarucci et al., 1998; Borsody and Weiss, 2005). Bilateral subdiaphragmatic vagotomy prevents sickness manifestations and activation of the NTS, hypothalamus and LC in rats treated IP with LPS or IL-1β (Bluthe et al., 1994; Bret-Dibat et al., 1995; Gaykema et al., 1995; Hansen and Krueger, 1997; Borsody and Weiss, 2005).

TNF-α-induced vagal immunosensory activity increases (Emch et al., 2000) or decreases (Emch et al., 2002) vagal motor activity. The fever and hyperalgesia caused by IP LPS are suppressed by the abdominal transection of vagal trunks, though this has a little effect on the febrile response to intravenous (IV) or intramuscular LPS. As subdiaphragmatic vagotomy blocks the induction of c-Fos protein in the rat hypothalamus and brainstem following IP injection of LPS, visceral afferent innervations are involved in the response to LPS. Conversely, vagotomy has a minimal effect on c-Fos protein induced by the IV administration of LPS (Wan et al., 1994). Moreover, LPS induces c-Fos activation of NTS neurons, which persists after cervical bilateral vagotomy (Hermann et al., 2001). Nonetheless, these blockages of CNS c-Fos activation are controversial. Neurons from the abdominal region or vagus efferent fibers (perhaps those within celiac branches, which transport LPS from the peritoneal cavity to the blood) may mediate the response to LPS *per se*. Therefore, vagotomy may eliminate responsive neurons, or it may restrict the amount of LPS escaping into systemic circulation, diminishing the systemic responses to LPS (e.g., c-Fos protein induction in the CNS) (Lenczowski et al., 1997; Romanovsky et al., 2000).

Finally, IL-1β and TNF-α did not modify the frequency of discharge recorded from single fibers in a preparation of isolated superfused rat GC from vagal paraganglia (Mac Grory et al., 2010), despite the expression of cytokine receptor in vagal afferent fibers (Goehler et al., 1997). Furthermore, neither the basal nor hypoxia-induced discharge rate of vagal paraganglia are modulated by IL-1β, TNF-α or LPS, suggesting that these structures are not the afferent limb of an “immune reflex” (O'Connor et al., 2012) (Figure 1). Thus, the neural signals from immune chemosensory inputs should originate from other receptors, and the neural pathway of peripheral arterial chemoreceptors (i.e., the carotid body and its sensory ganglion) provides an interesting candidate.

## The stimulation of efferent vagus nerve in sepsis therapy

The role of the vagus nerve and its stimulation has been studied in systemic inflammation. Vagus nerve stimulation has an anti-inflammatory effect in endotoxemia and downregulates proinflammatory cytokine production in sepsis, decreasing the plasma levels of HMGB1 and improving survival in cecal ligation and puncture (CLP), a model of septic peritonitis. Unilateral cervical (Van Westerloo et al., 2005) or subdiaphragmatic vagotomy (Kessler et al., 2006) increases the plasma levels of pro-inflammatory cytokines, tissue damage and mortality in sepsis. Additionally, in septic rats, vagus nerve electrical stimulation attenuates and prevents hypotension (Song et al., 2008) and modulates coagulation activation and fibrinolysis (Van Westerloo et al., 2006), decreasing MOD. The therapeutic potential of vagal (cholinergic) efferent fibers to treat disorders characterized by cytokine dysregulation is reviewed elsewhere (Rosas-Ballina and Tracey, 2009; Tracey, 2009).

## Immunosensory signaling through carotid body chemoreceptors

Anatomically, the carotid body (CB) is the largest paraganglion in the body (Mascorro and Yates, 1980), and its sensory innervation occurs through the carotid/sinus nerve, the nerve endings of which establish abundant synapses with specialized GC (Verna, 1997). The cell bodies of sensory pseudo-monopolar neurons innervating the CB are mainly located in the petrosal ganglion (Kalia and Davies, 1978; Berger, 1980). Afferent carotid/sinus nerve fibers establish the first synapsis in the NTS, at the CNS, in the same way as vagal afferents (Figure 1) (Donoghue et al., 1984; Finley and Katz, 1992).

Due the rich vascularization and abundant chemosensory innervations, we recently proposed that the CB is a peripheral sensor for the presence of immunogenic agents in the blood. Although the canonical LPS receptor, Toll-like receptor (TLR)-4 (Fernandez et al., 2011), and TNF-α receptors are functional (Fernandez et al., 2008, 2011), TNF-α does not modify the chemosensory discharge recorded under normoxic conditions from the carotid nerves of *in vitro* perfused and superfused cat CB. Nevertheless, TNF-α reduces in a dose-dependent manner the hypoxia-induced enhanced frequency of chemosensory discharge (Fernandez et al., 2008) but enhances the [Ca^2+^]_i_ response to acute hypoxia of dissociated GC. The increase is significantly larger in cells from the CB of rats exposed to chronic hypoxia or chronic intermittent hypoxia (Lam et al., 2008, 2012). Finally, TNF-α receptor expression in human and mouse carotid bodies was observed using microarray analysis, though the technique did not detect TNF-α (Mkrtchian et al., 2012).

Glomus (type I) cells from rat CB express both IL-1 receptor type I (Wang et al., 2002) and IL-6 receptor α (Wang et al., 2006). *In vitro*-cultured GC respond to IL-1β with depolarization and a transient rise in [Ca^2+^]_i_. Furthermore, IL-1β significantly increases carotid/sinus nerve chemosensory discharge in anesthetized rats (Shu et al., 2007), though the extracellular administration of IL-6 induces a rise in [Ca^2+^]_i_ and catecholamine release from *in vitro*-cultured GC (Fan et al., 2009). In addition, the IP administration of IL-1β in rat GC up-regulates both IL-1 receptor type I and tyrosine hydroxylase (Zhang et al., 2007).

We have reported a significant and maintained increase in basal chemosensory discharge after IV infusion of LPS in cats (Fernandez et al., 2008). Additionally, LPS increases CB TNF-α expression in rats (Fernandez et al., 2011), though we have not assessed *in situ* TNF-α administration. Neither IL-1β nor IL-6 expression in the CB during sepsis has been reported, but systemic pro-inflammatory cytokines could reach the CB because of their extensive vascularization (Verna, 1979). Thus, increased basal CB chemosensory activity could be due to either IL-1β or IL-6 stimulation. IL-1β appears to mimic the responses of the CB to hypoxia (i.e., evokes GC [Ca^2+^]_i_ oscillations and induces the expression of hypoxia-inducible factor (HIF), a transcription factor essential for the maintenance of normal CB activity during hypoxia) and may, therefore, act in an autocrine manner to enhance the peripheral chemoreceptor drive during systemic inflammation.

In septic cats, CB sensitivity to both stimulant (hypoxia and nicotine) and depressant (hyperoxia) stimuli is decreased (Fernandez et al., 2008). *In vitro* experiments have shown that TNF-α reduces the hypoxia-induced enhanced frequency of chemosensory discharge in a dose-dependent manner. Thus, TNF-α modulates CB chemosensory activity, perhaps by inducing the GC to release an inhibitory transmitter, such as dopamine. This fact has not yet been tested.

Microarray analyses of human and mouse CBs have shown increased expression of many other genes involved in immune and inflammatory responses. In addition to the above-mentioned pro-inflammatory cytokines, the transcripts of nuclear factor (NF)-κB, IL-10R (but not IL-10), and HMGB-1 have also been found in human and mouse CBs (Mkrtchian et al., 2012). In addition, the IP administration of LPS in rats decreased the cytosolic fraction of IκBα in the CB, evoking subsequent NF-κB p65 translocation into the GC nucleus, which resulted in gene expression, i.e., TNF-α up-regulation (Fernandez et al., 2011).

The expression of pro-inflammatory cytokines and their receptors in the CB suggests that those cytokines may activate chemosensory neurons, even in the absence of sepsis syndrome, exerting a tonic control of cardiorespiratory, endocrine, autonomic, and/or immune functions. Consequently, pro-inflammatory cytokines, through GC membrane receptors, may modify chemosensory activity reaching the NTS, modulating specific components of the systemic inflammatory response (Figure 1).

Pentobarbitone-anesthetized cats treated IV with LPS showed tachypnea, tachycardia, and hypotension, symptoms comparable to patients with severe sepsis and septic shock. Of note, bilateral section of the carotid and aortic nerves prevented increased respiratory rates (Fernandez et al., 2008). Additionally, LPS enhances tonic CB chemosensory activity (i.e., the frequency of chemosensory discharges), whereas LPS reduces CB responsiveness to both transient excitatory (hypoxia and nicotine) and depressant (F_i_O_2_ = 100%) stimuli (Fernandez et al., 2008). The reduced ventilatory responses to moderate and severe hypoxia observed in cats are similar to those observed in rats and in unanesthetized newborn piglets subjected to *E. coli* endotoxin infusion (McDeigan et al., 2003). This reduction in ventilatory responses is mediated –at least in part– by the inhibitory effect of endothelial nitric oxide (NO) on respiratory control mechanisms (Ladino et al., 2007).

Hyperoxia, which, in fact, reduces CB chemosensory activity (Fernandez et al., 2003), is associated with higher plasma levels of IL-6, IL-10 and TNF-α, a greater number of infected biological samples, and mortality in CLP-induced septic rats (Rodriguez-Gonzalez et al., 2014). Consequently, the withdrawal of carotid chemo/baro-sensory function modifies the inflammatory response during sepsis syndromes through a network of neural, humoral and cytokine elements.

The activation of DVC neurons did not require intact vagal pathways, suggesting that peripherally generated TNF-α could act either directly on these neurons –because DVC displays the attributes of CVOs– (Hermann et al., 2001) or, more likely, through another neural afferent pathway. Bilateral vagotomy does affect c-Fos expression in the NTS (Hermann et al., 2001). However, we found that bilateral carotid/sinus neurotomy after IP administration of LPS suppresses both the LPS-induced increase in the number of c-Fos-positive neurons of the NTS –with no significant changes in AP c-Fos immunoreactivity– and the increased levels of plasma cortisol (Reyes et al., 2012). Accordingly, we suggest that the neural signals provided by peripheral receptors that are distinct from vagal paraganglia –such as arterial carotid chemoreceptors, the function of which is intact after bilateral cervical vagotomy– produce prominent CNS manifestations of endotoxemia. These findings are particularly interesting because the CB induces –at least in part– an endocrine response to LPS by acting as an intermediate in the activation of the NTS by pro-inflammatory cytokines.

## Carotid body stimulation as a target for sepsis therapy: sympathetic activation and glucocorticoids release

The analysis of heart rate variability (HRV) gives a clear idea about the autonomic (sympathetic/parasympathetic) regulation of cardiorespiratory function. Decreased HRV is consistent with the pathogenesis of MOD; in fact, endotoxemic patients show decreased HRV (Godin et al., 1996; Rassias et al., 2005). Moreover, septic patients have an impaired sympatho-vagal balance that is characterized by a sustained sympatho-excitation accompanying hypotension (Barnaby et al., 2002), and chemo- and baro-denervation accelerates the drop in blood pressure (Vayssettes-Courchay et al., 2005). Finally, decreased parasympathetic activity is an excellent predictor of risk of death in patients with sepsis (Chen et al., 2008). Altogether, these data suggest that reflex arcs involved in maintaining the autonomic balance are altered during sepsis.

Carotid body stimulation provokes a wide array of cardiopulmonary and autonomic reflexes as well as endocrine responses (e.g., plasma release of catecholamines and cortisol) (Fitzgerald, 2014). In particular, chemoreflexes are important modulators of sympathetic activation (Abboud and Thames, 1983), and peripheral chemoreceptor activation elicits respiratory and cardiovascular effects and a sympatho-excitatory response (Alanis et al., 1968; Montarolo et al., 1976). Thus, tonic activation of carotid chemoreceptors during sepsis may also contribute to high levels of sympathetic activity (Kara et al., 2003). However, the administration of 100% O_2_ decreases the heart rate, blood pressure and central sympathetic outflow (Kara et al., 2003), and hyperoxia-induced CB chemosensory activity withdrawal is associated with higher plasma levels of pro-inflammatory cytokines and mortality in septic rats (Rodriguez-Gonzalez et al., 2014).

In addition, in anesthetized, paralyzed, ventilated and maintained normocapnic mongrel dogs, hypoxic hypoxia (the natural stimulus of CB chemoreceptors) increases the adrenal cortisol secretion rate, and surgical CB and/or aortic body deafferentation attenuates cortisol response (Raff et al., 1982). Thus, the CB exerts the main chemoreceptor influence on cortisol secretion during hypoxia. Interestingly, as mentioned above, bilateral carotid/sinus neurotomy attenuated the LPS-induced cortisol response in septic rats (Reyes et al., 2012).

Consequently, as a therapeutic target, the electrical stimulation of CB chemoreceptors could modify the inflammatory response during sepsis syndromes through a network consisting of neural (sympathetic activation), humoral (glucocorticoid secretion) and, as a consequence, cytokine elements.

## Conclusions

The knowledge of immunometabolic and neurophysiological mechanisms and the pathophysiology of sepsis progression to produce organ dysfunction and death have helped in the improvement of current therapies and in identifying new pharmacological therapeutic targets.

Traditionally, the autonomic nervous system coordinates the fine-tuning of the cardiorespiratory relationship, maintaining appropriate metabolite and oxygen delivery to tissues. Several reflex arcs, such as arterial baroreflexes, central chemoreflexes, peripheral arterial chemoreflexes, and pulmonary stretch reflexes, maintain the autonomic (sympathetic-parasympathetic) equilibrium. Consequently, the interactions among those reflexes are clinically interesting because the pathophysiological over-reaction of a single reflex, which occurs in several disorders, may cause the suppression of the opposite reflex responses.

An increasing body of evidence obtained by us and other researchers shows that CB reflexes not only serves as a chemoreceptor for respiratory reflex responses but also as a sensor for immune status (Zapata et al., 2011) and as a modulator of autonomic balance, tending to coordinate the cardiorespiratory interplay devoted to maintaining oxygen homeostasis in different pathologies.

In summary, CB stimulation increases sympathetic activity and glucocorticoid release. Thus, increased basal CB chemosensory activity during sepsis could be responsible, at least in part, for the observed increase in plasma epinephrine and cortisol levels in septic patients. The resulting increased plasma anti-inflammatory mediators could modulate pro-inflammatory cytokine expression in cytokine-producing cells, thereby modifying systemic inflammation and sepsis resolution. The electrical stimulation of the carotid/sinus nerve is a potential therapeutic approach, though not yet assessed, for sepsis therapy.

### Conflict of interest statement

The authors declare that the research was conducted in the absence of any commercial or financial relationships that could be construed as a potential conflict of interest.
